# The tetratricopeptide repeat-containing protein slow green1 is required for chloroplast development in *Arabidopsis*

**DOI:** 10.1093/jxb/ert463

**Published:** 2014-01-13

**Authors:** Zhihong Hu, Fan Xu, Liping Guan, Pingping Qian, Yaqiong Liu, Huifang Zhang, Yan Huang, Suiwen Hou

**Affiliations:** ^1^Key Laboratory of Cell Activities and Stress Adaptations, Ministry of Education, School of Life Sciences, Lanzhou University, Lanzhou 730000, PR China; ^2^National Key Laboratory of Plant Molecular Genetics, Institute of Plant Physiology and Ecology, Shanghai 200032, PR China

**Keywords:** Albino, Arabidopsis thaliana, chloroplast development, proplastid to chloroplast transition, slow greening, tetratricopeptide repeat-containing protein.

## Abstract

A new gene, *SG1*, was identified in a slow-greening mutant (*sg1*) isolated from an ethylmethanesulphonate-mutagenized population of *Arabidopsis thaliana*. The newly formed leaves of *sg1* were initially albino, but gradually became pale green. After 3 weeks, the leaves of the mutant were as green as those of the wild-type plants. Transmission electron microscopic observations revealed that the mutant displayed delayed proplastid to chloroplast transition. The results of map-based cloning showed that *SG1* encodes a chloroplast-localized tetratricopeptide repeat-containing protein. Quantitative real-time reverse transcription–PCR data demonstrated the presence of *SG1* gene expression in all tissues, particularly young green tissues. The *sg1* mutation disrupted the expression levels of several genes associated with chloroplast development, photosynthesis, and chlorophyll biosynthesis. The results of genetic analysis indicated that *gun1* and *gun4* partially restored the expression patterns of the previously detected chloroplast-associated genes, thereby ameliorating the slow-greening phenotype of *sg1*. Taken together, the results suggest that the newly identified protein, SG1, is required for chloroplast development in *Arabidopsis*.

## Summary

A chloroplast-localized tetratricopeptide repeat-containing protein, SG1, was identified through a slow-greening mutant in *Arabidopsis*. *SG1* is required for proplastid to chloroplast transition and its mutation disrupted the transcriptions of chloroplast-related genes. It also genetically interacts with *GUN1* or *GUN4*.

## Introduction

The chloroplast is a crucial organelle in higher plants. It is essential for fixation of CO_2_ and also for biosynthesis of carbon skeletons, fatty acids, pigments, and amino acids from inorganic nitrogen (Staehelin, 2000). Plastids are generally believed to have originated from a unicellular photosynthetic bacterium, which was incorporated into a eukaryotic host cell (Dyall, 2004). During evolution, most of the genes encoded by the bacterial ancestor were transferred to the host nuclear genome. For example, the plastid genome of *Arabidopsis thaliana* encodes ~100 proteins; however, >2000 proteins are encoded by the nuclear genes that function in the chloroplast (Abdallah *et al.*, 2000; Richly and Leister, 2004; Cui, 2006). Consequently, normal plastid development depends on the coordination of nuclear and plastid signals. This coordination is accomplished by nuclear signals that regulate the expression of plastid-encoded and nuclear-encoded plastid proteins, and also by signals sent from the developing plastids to the nucleus.

Plastids send signals to the nucleus via retrograde signalling, which operates through four distinct signal transduction pathways that are dependent on tetrapyrrole biosynthesis, plastid gene expression, the plastid redox state, or reactive oxygen species (ROS) (Surpin *et al.*, 2002; Nott *et al.*, 2006; Kakizaki *et al.*, 2009). The identification and characterization of the *genomes uncoupled* (*gun*) mutants (*gun1*–*gun6*), in which the developmental status of plastids and the nuclear-encoded chloroplast genes are uncoupled, have considerably enhanced understanding of retrograde signalling (Susek *et al.*, 1993; Mochizuki *et al.*, 2001; Larkin *et al.*, 2003; Woodson *et al.*, 2011). An example of this uncoupling is the abnormally high expression level of light-harvesting chlorophyll *a*/*b*-binding protein 1 (*LHCB1.1*) in *gun* mutants when chloroplast development is blocked by the herbicide norflurazon {4-chloro-5-(methylamino)-2-[3-(trifluoromethyl) phenyl]-3-(2H)-pyridazinone} (Susek *et al.*, 1993; Mochizuki *et al.*, 2001).

The process of chloroplast biogenesis is a complex plastic process, involving the interaction of environmental, cellular, and temporal factors (Pogson and Albrecht, 2011). The most influential environmental factor is light—in the absence of light, proplastids change into etioplasts (Robertson and Laetsch, 1974). Cellular factors include factors inside and outside the plastids. Most of the identified cellular factors are chloroplast-localized proteins involved in protein import, chloroplast gene transcription, RNA maturation, and protein translation and assembly (Pogson and Albrecht, 2011). Temporal factors such as embryo maturation can also influence chloroplast development (Albrecht *et al.*, 2008; Kim *et al.*, 2009; Pogson and Albrecht, 2011).

In flowering plants, the development and activity of chloroplasts differ between cotyledons and true leaves. In cotyledons, plastids partially develop during embryogenesis; however, their development is arrested during seed maturation and dormancy. Cotyledons serve primarily as storage organs until the seedling becomes autotrophic; the cotyledons may then develop chloroplasts (Mansfield and Briarty, 1996; Charuvi *et al.*, 2012). In contrast, the chloroplasts of true leaves differentiate directly from the proplastid present in the shoot apex, and their primary function is photosynthesis (Charuvi *et al.*, 2012). In developed leaves, chloroplasts are further propagated by fission, similar to that observed in bacteria (Pyke, 1997; Leon *et al.*, 1998; Pogson and Albrecht, 2011). These differences have been examined in mutants having chloroplast defects restricted either to the cotyledons or to the true leaves. For instance, the *snowy cotyledon* (*sco*) mutant group has chlorotic or bleached cotyledons but green true leaves (Albrecht *et al.*, 2006, 2008, 2010; Shimada *et al.*, 2007). Conversely, *immutans* (*im*) and *variegated2* (*var2*) mutants have green cotyledons but chlorotic true leaves (Aluru *et al.*, 2006; Kato *et al.*, 2007; Liu *et al.*, 2010).

In the past decade, studies of chloroplast development mutants have enhanced our understanding of the transition from proplastids to chloroplasts within true leaves. A useful and well-characterized mutant of *Arabidopsis*, *var2*, has green cotyledons and true leaves with green and white areas. The phenotype is the result of deactivation of a metalloprotease, FtsH2. The chloroplasts in the green sectors are normal; however, the white sectors contain undifferentiated plastids, indicating the blockage of proplastid to chloroplast transition (Kato *et al.*, 2007; Sakamoto *et al.*, 2009). The occurrence of such variegated mutants has also been reported in maize (*Zea mays*) and tomato (*Solanum lycopersicum*) (Han *et al.*, 1992; Carol and Kuntz, 2001). In *Arabidopsis*, the *delayed greening1 mutant* (*dg1*) has a delayed-greening phenotype, caused by postponed chloroplast development (Chi *et al.*, 2008). Many albino mutants have also been reported, for example *csr1-1* in *Z. mays* and *atecb2* in *Arabidopsis* (Asakura *et al.*, 2004; Yu *et al.*, 2009). Most of these mutations are lethal, because they prevent the development of mature chloroplasts.

In the present study, a tetratricopeptide repeat (TPR)-containing protein, slow green1 (SG1), was identified that affects chloroplast development in *Arabidopsis*. The *sg1* mutant had disturbed expression of chloroplast-related genes and displayed delayed chloroplast differentiation. The findings suggest that SG1 is required for chloroplast development in *Arabidopsis*.

## Materials and methods

### Plant materials and growth conditions

*Arabidopsis* ecotype Columbia (Col) was used as the wild type (WT). T-DNA insertion lines SALK_046229C and SALK_026339 were obtained from the ABRC (Ohio State University). The seeds of *gun1-1*, *gun4-1*, and *gun5-1* were kindly provided by Professor Enrique Lopez Juez (University of London, UK). All plants were grown at room temperature (22–25 °C) under long-day conditions (16h light/8h dark). To inhibit photoinhibition, *sg1* was grown under 80 mmol m^–2^ s^–1^ white light. For phenotype identification, all plants were grown under conditions similar to that used for *sg1*. Otherwise, the plants were grown under 125 mmol m^–2^ s^–1^ white light.

### Mutant isolation and mapping

The *sg1* mutant was isolated from an ethylmethanesulphonate (EMS)-mutagenized M_2_ population with a Col background. The *SG1* locus was mapped by using individuals of an F_2_ population derived from a cross between *sg1* and WT L*er* (Landsberg *erecta*). After PCR amplification, genetic markers were scored as simple sequence length polymorphism (SSLPs) or cleaved amplified polymorphic sequences (CAPSs) (Michaels and Amasino, 1998). The markers and restriction enzymes used to reveal the polymorphisms are detailed in Supplementary Tables S3 and S4 available at *JXB* online. MIE15 was a CAPS marker cleaved by the *Mse*I-restricted enzyme. Map distances were calculated according to Kosambi (1943).

### Transmission electron microscopy

Immediately after harvest, the sixth leaves from plants at different growth stages were cut into small pieces, and fixed in 2.5% glutaraldehyde in phosphate buffer (pH 7.4) for 4h at 4 °C. The samples were rinsed and incubated in 1% OsO_4_ for 12h at 4 °C. Then the samples were rinsed with phosphate buffer (pH 7.0), infiltrated with a graded series of epoxy resin in epoxy-propane, and then embedded in Epon 812 resin. Thin sections (~50nm) were obtained by using an ultramicrotome (Leica). The sections were stained in 2% uranyl acetate (pH 5.0), followed by 10mM lead citrate (pH 12), and viewed under a transmission electron microscope (JEM-1230). All images in this study were processed using Adobe Photoshop and Image J software.

### Complementation test and overexpression of *SG1*

For the complementation test, the region between ~1500bp upstream of *SG1* and the entire genomic fragment of *SG1* was PCR-amplified by using the primers SG1-P1F (*ACG CGT* CGA CGT CTT GGC CTT TTA GTA GTT TAA TG) and SG1-P1R (GG *GGT ACC* GTT CTC CTC ACT ACC AC), and cloned into the binary vector pCAMBIA1300 by using the *Sal*I and *Kpn*I enzymes (underlined regions indicate the introduced *Sal*I and *Kpn*I sites, respectively). For overexpression of *SG1*, the full-length genomic DNA of *SG1* (introns are absent in *SG1*) was amplified by using the primers SG1-P2F (GC *TCT AGA* GCA TGA TTT CGT CTC TCT CAG) and SG1-P1R, and cloned into the binary vector pCAMBIA1300-GFP (green fluorescent protein) by using the *Xba*I and *Kpn*I enzymes. The resulting plasmids *pSG1::SG1::GFP* and *p35S::SG1::GFP* were transferred into *Agrobacterium tumefaciens* strain GV3101 (Koncz and Schell, 1986), and transformed into *sg1* or WT plants by using the floral dip method (Clough and Bent, 1998). Transformed plants were selected on Murashige and Skoog (MS) medium containing 25mg l^–1^ hygromycin.

### Subcellular localization of GFP proteins

The constructed *p35S::SG1::GFP* plasmid was transformed into *Arabidopsis* protoplasts to observe the transient expression of the fusion protein. Meanwhile the *p35S::GFP* vector was transformed as a control. The procedures for protoplast isolation and plasmid transformation were described by Kim and Somers (2010). The transformed protoplasts were observed by using confocal microscopy. A 488nm argon ion laser line was used for excitation of GFP and chlorophyll, while 505–515nm and 650nm emission filters were used for simultaneously capturing GFP and chlorophyll fluorescence, respectively, by using an Olympus FV1000MPE2 confocal microscope.

### Chlorophyll detection

Total chlorophyll was determined in triplicate according to the method described by Lichtenthaler and Wellburn (1983). Extracts were obtained from the sixth leaves or seedlings at different growth stages. Approximately 0.2g of fresh tissue was homogenized in 5ml of 80% acetone for 12h in darkness. Spectrophotometric quantification was carried out in a Gene Quant spectrophotometer (GE Healthcare), using the following calculations: Chl *a*=12.21×A_663_–2.81×A_646_, and Chl *b*=20.13×A_646_–5.03×A_663_ (μg ml^–1^).

### Real-time reverse transcription–PCR

Total RNA was extracted from 0.5g of plant tissue by using the E.Z.N.A Plant RNA Kit (Omega) according to the manufacturer’s instructions, with the addition of an RNase-free DNase I treatment (Omega). The cDNAs were synthesized from 1 μg of total RNA using the Prime Script™ RT Reagent Kit (Perfect Real Time; Takara). All of the quantitative real-time reverse transcription–PCR (qRT–PCR) measurements were performed using an MX 3000 Real-time PCR system (Stratagene) with SYBR Premix Ex Taq (Takara, Japan), according to the manufacturer’s instructions. The housekeeping gene *β-tubulin* was used as a normalization control. The relative expression was calculated by using the formula 2^–ΔΔCt^. All the experiments were performed for each biological replicate. The primer sequences for qRT–PCR are provided in Supplementary Table S5 available at *JXB* online.

### Protein extraction and SDS–PAGE

Seedlings or different growth stage leaves harvested were immediately frozen in liquid nitrogen and pulverized. Total proteins were extracted in extraction buffer (0.1% Triton X-100, 0.1% SDS, 0.01M EDTA, 0.01M β-mercaptoethanol, 0.05M Na_2_HPO_4_, pH 7.0). The homogenates were centrifuged at 12 000rpm for 15min at 4 °C. The protein concentrations of the supernatants were determined by using western blotting of β-tubulin. Total proteins were separated by 12% SDS–PAGE. For each experiment, a minimum of three independent replicates were performed.

## Results

### Identification of a slow-greening mutant

**Fig. 1.**
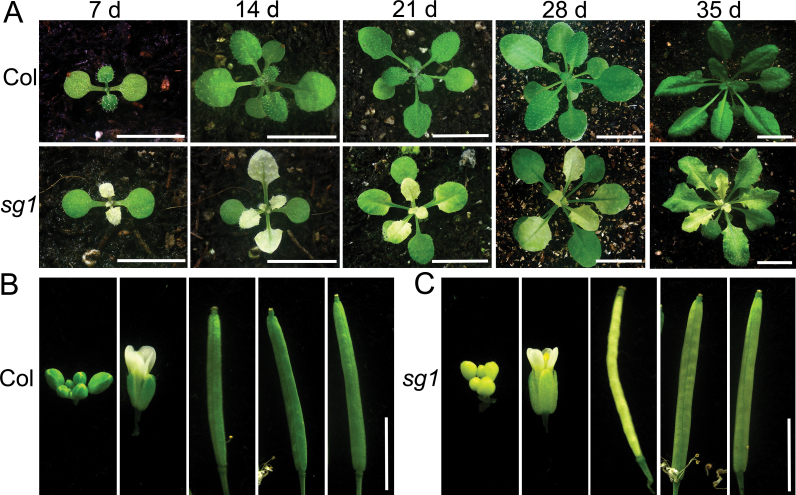
The phenotypes of the *sg1* mutant. (A) Seedlings of the *sg1* mutant and WT (Col) grown for 1–5 weeks in soil. (B, C) Inflorescences and young siliques of the Col and *sg1* mutant. d, days after germination. Bars=1cm in (A); 0.5cm in (B, C).

To identify the genes involved in chloroplast development, a slow-greening mutant, designated *sg1*, was isolated from an EMS-mutagenized population of *Arabidopsis*. The initial rosette leaves of *sg1* were completely albino, but gradually became green (Fig. 1A). At ~3 weeks post-emergence, the leaves of the mutant were as green as those of the WT (Fig. 1A). The slow-greening phenotype was apparent in other newly formed organs of *sg1*, including the stems, inflorescences, and siliques. The young inflorescences and siliques of *sg1* were white or pale green, and became green as they matured (Fig. 1B, C). These observations are consistent with a pigment deficiency in *sg1*, and therefore the levels of chlorophyll *a* and chlorophyll *b* were measured at different growth stages of leaf development. The sixth leaves of 3-, 4-, and 5-week-old *sg1* mutant plants (exhibiting albino, pale-green, and green leaves, respectively) and the corresponding Col leaves were used. Consistent with their phenotypes, the chlorophyll contents increased as the leaves turned green; when the sixth leaves of 5-week-old *sg1* mutants became green, the chlorophyll contents were markedly higher than those of the albino leaves, but lower than those of the WT (see Supplementary Table S1 available at *JXB* online). Over time, the chlorophyll contents of the mutant and wild-type leaves became comparable. At all growth stages, the mutant plants were smaller than the WT (Fig. 1A).

Many chloroplast-development mutants of *Arabidopsis* can grow well when supplied with sucrose as a carbon source (Koch, 1996; Chi *et al.*, 2008; Yu *et al.*, 2009); further, they may show abnormal embryo development (Uwer *et al.*, 1998; Apuya *et al.*, 2001; Kobayashi *et al.*, 2007). To investigate whether SG1 is involved in chloroplast development, *sg1* seedlings were grown on MS medium without or with 2% sucrose, and the *sg1* embryogenesis of *sg1* homozygotes was observed. It was determined that sucrose partially alleviated the albino phenotype of *sg1*, and also that embryogenesis of *sg1* in heterozygote plants was delayed (see Supplementary Fig. S1 available at *JXB* online). The results suggest that *sg1* affects chloroplast development during the early stages of seedling growth.

### Differentiation of proplastids into chloroplasts

**Fig. 2.**
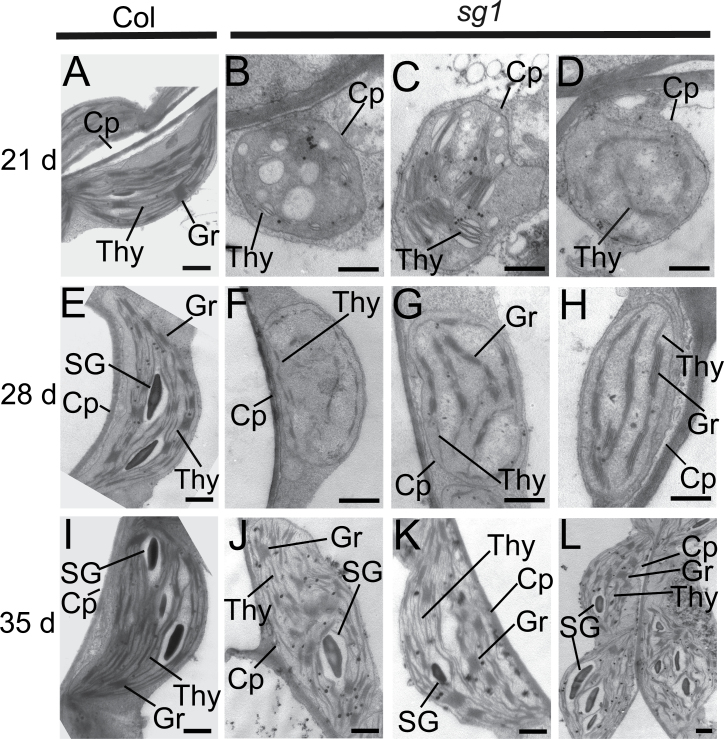
Transmission electron micrographs of chloroplasts from the sixth leaves of 3- to 5-week-old Col and *sg1* plants. (A, E, I) Chloroplast structures from the wild-type plants. (B–D), (F–H), and (J–L) Chloroplast structures from the *sg1* mutant plants. Cp, chloroplast; SG, starch grain; Thy, thylakoid; Gr, grana thylakoids. Bars=1 μm.

The delayed-greening phenotype of *sg1* implies defective chloroplast development. Transmission electron microscopy was used to examine the chloroplast ultrastructures of the sixth leaves of 3-, 4-, and 5-week-old mutant plants (exhibiting albino, pale-green, and green leaves, respectively). In wild-type plants grown under normal conditions, the proplastid to chloroplast transition occurs at the shoot apical meristem during the early stages of development (Charuvi *et al.*, 2012). Thus, in the present study, the chloroplasts of WT leaves at the same developmental stages were already differentiated and crescent-shaped, and contained well-developed thylakoid membranes with grana stacks (Fig. 2A, E, I). Starch grains were lacking in the chloroplasts of 3-week-old WT plants (Fig. 2A), but were present in the chloroplasts of 4- and 5-week-old plants (Fig. 2E, I). The albino leaves of 3-week-old *sg1* seedlings contained few well-developed crescent-shaped chloroplasts, but many smaller, abnormal, irregularly shaped chloroplasts, similar to proplastids (Fig. 2B–D). These abnormal chloroplasts could be classified into three types according to their morphologies. The first type was rounded and highly vacuolated, with almost no thylakoid membrane, and appeared undifferentiated (Fig. 2B). The second type had fewer vacuoles and easily observable thylakoid membranes (Fig. 2C), possibly representing an intermediate form between the proplastid and chloroplast. The third type had discontinuous thylakoid membranes, resembling chloroplasts at an early stage of development (Fig. 2D). The pale-green leaves of 4-week-old *sg1* seedlings contained differentiated chloroplasts that were smaller and had fewer thylakoid membranes than did chloroplasts of WT seedlings at the same growth stage (Fig. 2F–H); these chloroplasts resembled WT chloroplasts at an early stage of development. Some of the thylakoid membranes were discontinuous and some were arranged as grana stacks (Fig. 2F–H). Similar to the WT, the green leaves of 5-week-old *sg1* plants contained chloroplasts with well-developed thylakoid membranes and grana stacks, and well-developed starch grains (Fig. 2J–L). The conversion of proplastids to chloroplasts in *sg1* occurred over a period of ~3 weeks. These results suggest the delayed transition of proplastids to chloroplasts in *sg1*, consistent with a slow-greening phenotype.

### Gene cloning and complementation of *sg1*

To verify whether *sg1* is a nuclear recessive mutant, the M_3_ generation families of *sg1* were crossed reciprocally with wild-type plants. Plants in the F_1_ generations of *sg1* (♂)×Col (♀) and Col (♂)×*sg1* (♀) were as green as the WT. The offspring of F_1_ plants from both of the crosses segregated in a 3:1 ratio (see Supplementary Table S2 available at *JXB* online). These results suggest that *sg1* is a single recessive gene mutation with nuclear inheritance.

**Fig. 3.**
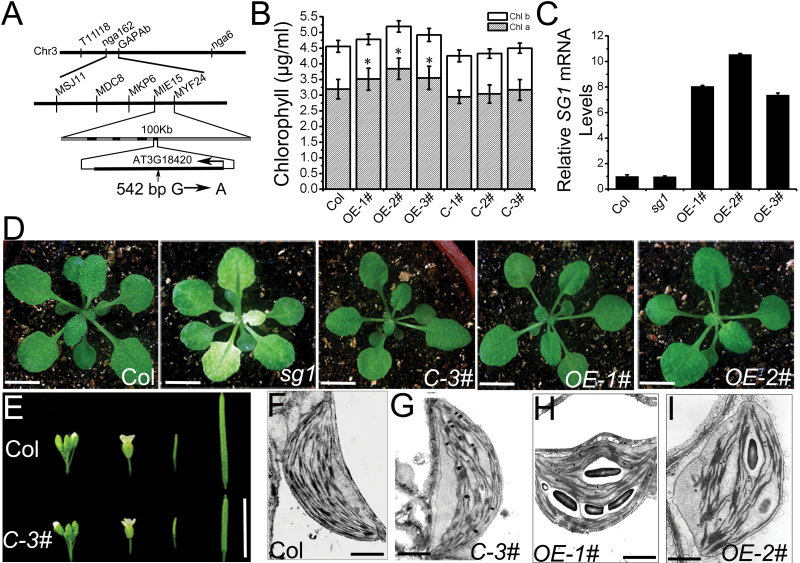
Identification of the *SG1* gene. (A) Map-based cloning, with the locations of molecular markers and mutation site indicated. (B) Chlorophyll concentration in mature rosette leaves of 4-week-old Col and overexpressing and complemented plants. (C) qRT–PCR analysis of *SG1* transcription levels in *sg1* and differently overexpressing transgenic lines with a Col background. (D) Seedlings of *sg1* mutants, complemented (*C-3#*), and overexpressing transgenic plants (*OE-1#* and *OE-2#*) grown for 4 weeks in soil. (E) Inflorescences and siliques of complemented plants (*C-3#*). (F–I) Transmission electron micrographs of chloroplasts in 4-week-old leaves from Col, the complemented (*C-3#*), and overexpressing transgenic plants (*OE-1#* and *OE-2#*). Bars=0.5cm in (D, E); 1 μm in (F–I). Values represent the mean±SD of three independent experiments. Asterisks denote significant differences (*P* < 0.05).

A map-based cloning approach was used to identify the mutated gene, by crossing *sg1* with the Landsberg *erecta* (L*er*) ecotype of *Arabidopsis*, to generate an F_2_ mapping population. SSLP markers (see Supplementary Table S3 available at *JXB* online) were selected for primary determination of the linkage group. On the basis of 21 F_2_ plants, it was concluded that *SG1* was located on the upper arm of chromosome 3, in the interval between the markers NGA162 (7.24%) and GAPAb (16.24%) (Fig. 3A). Additional InDel markers and a CAPS marker (Fig. 3A; Supplementary Table S4 available at *JXB* online) were used to refine the position of *SG1*. On the basis of 146 F_2_ plants, the location of *SG1* was narrowed down to an ~110kb region between the markers MIE15 and MYF24. In this region, only four genes encoding proteins predicted to be involved in chloroplast localization were identified, namely *AT3G18230*, *AT3G18270*, *AT3G18390*, and *AT3G18420*. Sequence analysis of the open reading frames of these four candidate genes revealed the existence of a single G to A mutation in base pair 542 from the start codon ATG of *AT3G18420* genomic DNA; this mutation caused a conversion of arginine to lysine in amino acid 181 of SG1 protein (Fig. 3A). The expression level of SG1 in the *sg1* mutant was determined, and it was shown that the G to A substitution did not affect mRNA accumulation (Fig. 3C). To confirm that the *SG1* gene is *AT3G18420*, the *sg1* mutant was genetically complemented with the full-length *AT3G18420* cDNA under control of the promoter (1500bp upstream of the open reading frame) of *AT3G18420*. A total of 22 T_1_ transgenic plants were screened for *pSG1::SG1* with a *sg1* background. Subsequent phenotypic observations confirmed that the complemented mutants had WT traits (Fig. 3D). Further, the chlorophyll contents of 28-day-old rescued transgenic plants were similar to those of the WT (Fig. 3B), and ultrastructural examination of the chloroplasts from the sixth leaves of these plants revealed that they were well developed and similar to those of the WT (Fig. 3F). These results indicate that *AT3G18420* can complement the chloroplast differentiation defects in the *sg1* mutant, thereby further suggesting that *AT3G18420* is responsible for the *sg1* phenotypes. Two T-DNA insertion lines were obtained from the Arabidopsis Biological Resource Center. The T-DNA insertion harboured 296bp upstream of the ATG translation start codon in the SALK_046229C line, and 74bp downstream of the TAA translation stop codon in the SALK_026339 line. The results of RT–PCR indicated that the *SG1* gene could be expressed in both T-DNA lines, without apparent effects on the chloroplasts.

To investigate further the function of *SG1* in chloroplast development, a plasmid containing the full-length cDNA of *AT3G18420*, under control of the *Cauliflower mosaic virus 35S* promoter (CaMV *35S*), was constructed, which was transformed to the WT. Thirty-one T_1_ overexpressing (*OE*) transgenic plants were screened, and no visible phenotypic effects were observed (Fig. 3C, D). The chlorophyll contents were measured and the ultrastructures of three *OE* lines were examined; it was determined that the content of chlorophyll *a* was slightly higher than that of WT plants (Fig. 3B). The chloroplasts of the *OE* lines were somewhat irregularly shaped, and had a slightly higher proportion of thylakoid membranes, especially in the grana; these characteristics may be responsible for the higher chlorophyll *a* content (Fig. 3B, H, I). The results indicate that *SG1* plays an important role in chloroplast development.

### Encoding of a conserved, widely expressed, chloroplast-localized TPR-containing protein by *SG1*

**Fig. 4.**
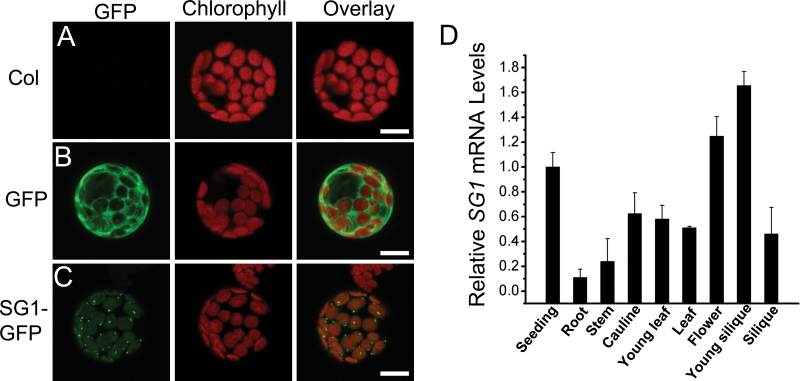
Subcellular localization and expression profiles of SG1. (A) Protoplast from a wild-type plant. (B, C) Protoplast transformed with *p35S::GFP* and *p35S::SG1::GFP*. Left to right: fluorescent image of GFP, chlorophyll autofluorescence, and merged image of GFP and chlorophyll autofluorescence. (D) Expression profiles of the *SG1* gene based on qRT–PCR analysis of *SG1* transcripts in various organs. Seedling, 10-day-old seedlings; root, roots from 10-day-old seedlings; stem, stem only, with all leaves and inflorescences removed; cauline, cauline leaves; young leaf, the sixth leaves of 5-week-old plants; leaf, mature rosette leaves of 5-week-old plants; flower, flower clusters; young silique, siliques at 1 week after fertilization; silique, green siliques at >1 week after fertilization. Bars=10 μm. Values represent the mean±SD of three independent experiments.

Analysis of the complete *Arabidopsis* sequence by using BLAST revealed that the nuclear genome contains a single copy of the *SG1* gene. The results of phylogenetic analysis and protein alignments indicated that SG1 was conserved during the evolutionary process; further, it shares significant identity with the (hypothetical) *Arabidopsis* proteins—AT2G37400, AT3G53560, AT5G02590, and AT3G09490—which are chloroplast lumen common family proteins (see Supplementary Fig. S2 available at *JXB* online). *SG1* encodes a putative polypeptide of 316 amino acids, with four TPR motifs (see Supplementary Fig. S2B available at *JXB* online). To confirm the subcellular localization of SG1, the transient expression of the *p35S-SG1-GFP* plasmid was examined in living *Arabidopsis* protoplasts. As a control, *Arabidopsis* protoplasts were transformed with a plasmid containing only GFP, under control of the *35S* promoter. The GFP signals were observed by using confocal laser-scanning microscopy. In the absence of transformation, only chlorophyll autofluorescence was detected (Fig. 4A). After transformation with the control vector, GFP signals accumulated ubiquitously in the cytosol (Fig. 4B). In contrast, in the *p35S-SG1-GFP*-transformed protoplasts, GFP signals were coincident with chlorophyll autofluorescence; further, some SG1–GFP fusion protein was observed accumulated as spots in the chloroplast, which may be important for its function (Fig. 4C).

Next, the expression profiles of *SG1* was examined. The mRNAs were isolated from different tissues of WT plants, and the *SG1* expression level was detected by using qRT–PCR. The housekeeping gene *β-tubulin*, the expression levels of which remain similar across different tissues, was used to normalize different samples. The expression level of *SG1* in 10-day-old seedlings was arbitrarily set to 1. It was determined that *SG1* was widely expressed in all *Arabidopsis* tissues (Fig. 4D). The highest expression levels were observed in young siliques and flower clusters, while the lowest expression level was observed in roots (Fig. 4D). *SG1* was ubiquitously expressed throughout the plant; however, its expression was preferentially associated with green tissues, particularly newly formed tissues. These results indicate that SG1 localizes to the chloroplast and exhibits ubiquitous expression.

### Disrupted expression levels of genes associated with chloroplast development, photosynthesis, or chlorophyll biosynthesis

**Fig. 5.**
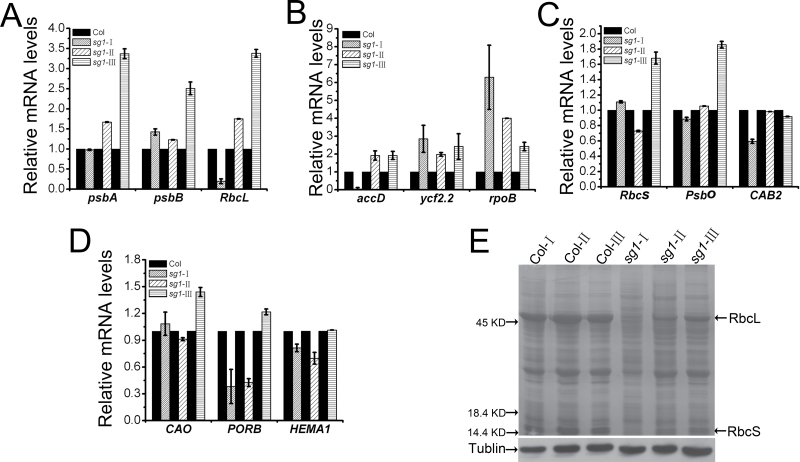
Transcript and protein analysis from leaves of *sg1* plants at different stages. (A–D) The expression levels of plastid-encoded polymerase (PEP) transcribed chloroplast genes, nucleus-encoded polymerase (NEP) transcribed chloroplast genes, nuclear-encoded chloroplast genes, and chlorophyll biosynthesis genes. (E) Total protein resolved by SDS–PAGE from leaves of *sg1* and corresponding wild-type plants at different growth stages. The terms *sg1*-I, *sg1*-II, and *sg1*-III refer to the sixth leaves that exhibited albino, pale-green, and green leaves in 3- to 5-week-old *sg1* mutants, respectively; Col-I, Col-II, and Col-III refer to the corresponding leaves in Col plants. Western blotting of β-tubulin was used to confirm equal loading.

The TPR or TPR-related motif-pentatricopeptide repeat (PPR) proteins are reported to be involved in chloroplast gene expression (Pfalz *et al.*, 2006; Chi *et al.*, 2008; Su *et al.*, 2012). Since abnormal chloroplast development was observed in *sg1*, the effect of the loss of *SG1* on the expression of chloroplast-related genes was investigated using qRT–PCR. The transcription levels of plastid-encoded polymerases (PEPs) and nucleus-encoded polymerases (NEPs), which transcribe chloroplast genes, nuclear-encoded chloroplast genes, and chlorophyll biosynthesis genes, were examined in the albino, pale-green, and green leaves of *sg1* plants, and also in the corresponding WT leaves (Fig. 5A–D). Three PEP genes, namely those encoding two members of photosystem II complexes (*psbA* and *psbB*) and a RuBisCO large subunit (*RbcL*), were selected (Fig. 5A). Three NEP genes were also selected, namely an *accD*, which encodes a carboxytransferase β subunit of the acetyl-CoA carboxylase (ACCase) complex, *ycf2.2*, which encodes a predicted chloroplast-localized ATP-binding protein, and *rpoB*, which encodes a chloroplast DNA-dependent RNA polymerase B subunit (Fig. 5B). Three nuclear-encoded chloroplast genes: RuBisCO small subunit (*RbcS*), light-harvesting chlorophyll *a*/*b*-binding protein (*CAB2*/*LHCB1.1*), and oxygen evolving polypeptide 1 (*PsbO*) were detected (Fig. 5C). Genes that are crucial to chlorophyll biosynthesis, namely *CAO* (encoding chlorophyllide-*a* oxygenase), *HEMA1* (encoding glutamyl-tRNA reductase 1), and *PORB* (encoding protochlorophyllide oxidoreductase B), were also selected for detection (Fig. 5D). The expression levels of *psbA*, *RbcL*, *accD*, *PsbO*, and *PORB* showed very similar tendencies. The expression levels of *RbcL*, *accD*, and *PORB* (especially *RbcL* and *accD*) were markedly lower in the albino leaves of mutant plants than in the WT; however, the expression levels of all five genes gradually increased as the leaves became green, to reach higher levels than those of the WT (Fig. 5A–D). Nevertheless, the expression of the *rpoB* gene showed the opposite expression pattern. Its expression level was markedly higher in albino leaves than in the leaves of the WT; further, the expression level gradually decreased as the leaves grew, but remained slightly higher in green leaves than in the leaves of the WT (Fig. 5B). The expression of *psbB*, *ycf2.2*, *RbcS*, and *CAO* was a little higher in *sg1* albino leaves than in the WT. These four genes and *HEMA1* (lower in albino leaves) decreased in pale-green leaves while they increased when the leaves turned green (Fig. 5A–D). The expression level of *CAB2* was lower in albino leaves than in the leaves of the WT; however, in pale-green and green leaves, it increased to approximately the same level as in the leaves of the WT (Fig. 5C). Thus, the expression levels of all four types of genes were affected in *sg1*. In addition, the soluble proteins were profiled in leaves of *sg1* mutant plants at different growth stages. It was determined that albino leaves of mutant plants contained significantly lower amounts of RbcL and RbcS (Fig. 5E). As the leaves became green, the amounts of these two proteins gradually increased (Fig. 5E). The increase in RbcL was in accordance with the transcript data. On the other hand, the expression level of *RbcS* did not change in the albino leaves, despite a significant decrease in the level of encoded protein. Furthermore, in the green leaves of *sg1*, the expression levels of *RbcL* and *RbcS* were higher than those in the corresponding WT leaves; however, the protein levels were lower than were those of the corresponding WT leaves. Therefore, *SG1* may also be involved in chloroplast protein biosynthesis and/or degradation.

### Genetic interaction of GUN1 and GUN4 with SG1

**Fig. 6.**
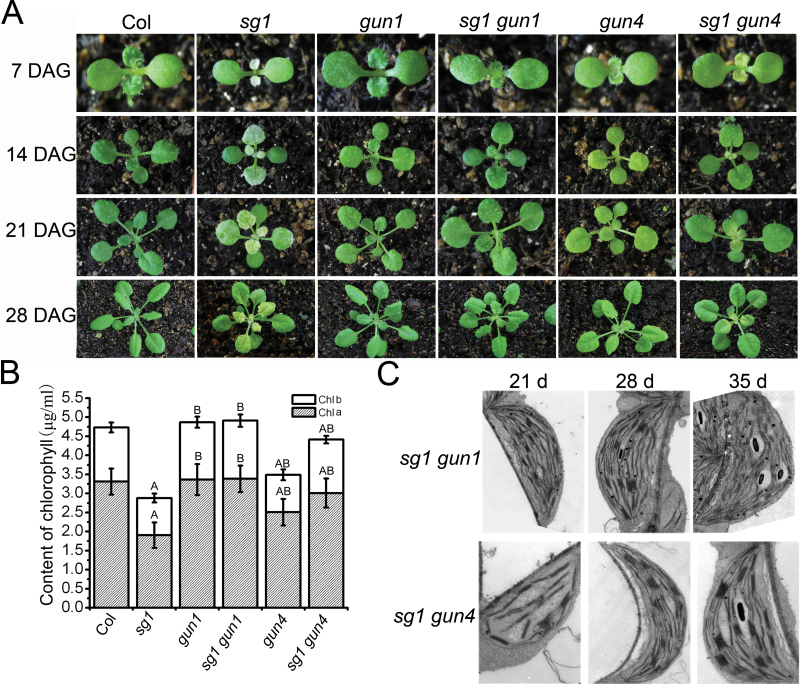
The *gun1* and *gun4* mutants alleviated the delayed greening phenotype of *sg1*. (A) Seedlings of Col, *sg1*, *gun1*, *sg1 gun1*, *gun4*, and *sg1 gun4* grown for 1–4 weeks in soil. (B) Chlorophyll concentrations of 2-week-old Col, *sg1*, *gun1*, *sg1 gun1*, *gun4*, and *sg1 gun4* seedlings with cotyledons removed. (C) Chloroplast structures of the sixth leaves from 3- to 5-week-old *sg1 gun1* and *sg1 gun4* plants. Bars=0.5cm in A; 1 μm in B. Values represent the mean±SD of three independent experiments. ^a^Significantly different from Col, *P* < 0.05;^ A^Significantly different from Col, *P* < 0.01;^ B^Significantly different from *sg1*, *P* < 0.01.

To investigate further the putative pathways in which SG1 may be involved in chloroplast development, *sg1 gun1* and *sg1 gun4* double mutants were constructed by crossing *sg1* with *gun1-1* and *gun4-1* mutants. Interestingly, both *sg1 gun1* and *sg1 gun4* double mutants alleviated the delayed-greening phenotype of *sg1* (Fig. 6A). The leaves of the *sg1 gun1* double mutant were of a similar green colour to the leaves of the WT plants (Fig. 6A). Further, this double mutant had no albino leaves, but the young buds and basal parts of the second and third inner leaves were slightly pale green (Fig. 6A). Similarly, few or no albino leaves were observed in *sg1 gun4*, and its mature leaves were completely green, unlike the pale-green phenotype of *gun4-1* (Mochizuki *et al.*, 2001; Larkin *et al.*, 2003) (Fig. 6A). In both of the double mutants, the newly formed inflorescences and siliques were of a similar green colour to those of WT plants. The chlorophyll contents of 2-week-old Col, *sg1*, *gun1*, *sg1 gun1*, *gun4*, and *sg1 gun4* seedlings were compared after removal of the cotyledons. It was determined that both of the double mutants had higher chlorophyll contents than did the *sg1* mutant (Fig. 6B). The *gun4* mutant showed chlorophyll defects; however, the chlorophyll contents of the *sg1 gun4* double mutant were considerably higher than were those of the *sg1* and *gun4* single mutants while they were lower than those of the WT. The chlorophyll contents of the sixth leaves (at different stages) of these plants were also measured, and concurring results were obtained (see Supplementary Table S1 available at *JXB* online).

The development of chloroplasts in the sixth leaves of 3-, 4-, and 5-week-old double mutants was observed. As expected, the transition from proplastid to chloroplast in *sg1 gun1* and *sg1 gun4* double mutants was significantly advanced relative to that in *sg1* plants, but slightly delayed in comparison with the WT (Figs 2A, E, I, 6C). In 3- to 5-week-old *sg1 gun1* double mutants, the development of chloroplasts in the sixth leaves was the same as in WT plants (Fig. 2A, E, I; Fig. 6C, upper panels). In the 3-week-old *sg1 gun4* double mutant, prophase chloroplasts formed, but the abundance of thylakoid membranes was lower than that in the corresponding WT plants (Fig. 2A, E, I; Fig. 6C, lower panels). Further, these chloroplasts were similar to those present in the sixth leaves of 4-week-old *sg1* plants (Fig. 2G, H; Fig. 6C, lower panels). Mature chloroplasts were present in the sixth leaves of 4-week-old and 5-week-old *sg1 gun4* plants (Fig. 6C, lower panels).

**Fig. 7.**
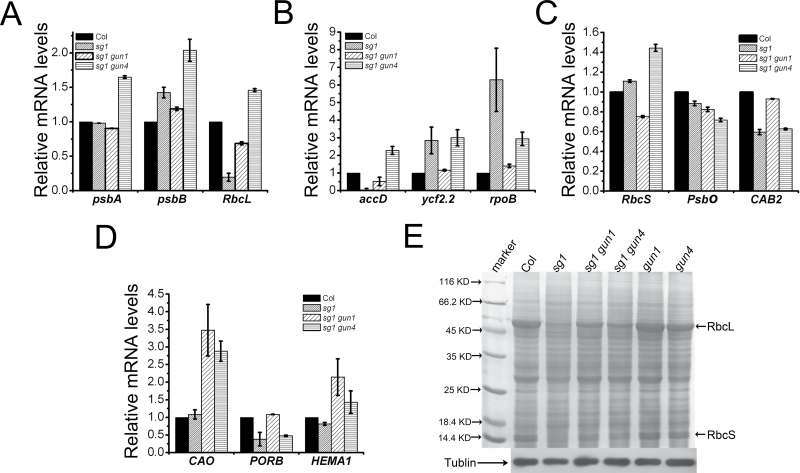
Transcript and protein analysis of 2-week-old Col, *sg1*, *sg1 gun1*, and *sg1 gun4*. (A–D) The expression levels of plastid-encoded polymerase (PEP) transcribed chloroplast genes, nucleus-encoded polymerase (NEP) transcribed chloroplast genes, nuclear-encoded chloroplast genes, and chlorophyll biosynthesis genes. (E) Total proteins resolved by SDS–PAGE from different genotype seedlings. The mRNA and total proteins were isolated from 2-week-old plants with cotyledons removed.

The expression levels of chloroplast-related genes were also quantified in 2-week-old Col, *sg1*, *sg1 gun1*, and *sg1 gun4* plants. To eliminate the influence of green cotyledons, these were removed before RNA isolation was performed. The results showed that both *gun1* and *gun4* mutations in the *sg1* background affected the expression of chloroplast-related genes compared with those in *sg1*. For example, both double mutants increased the expression levels of *RbcL* and *accD* (expressed at very low levels in *sg1*), and decreased the expression level of *rpoB* (expressed at much higher levels in *sg1*) (Fig. 7A–D). Also, the chlorophyll biosynthesis genes *CAO* and *HEMA1* were increased in both double mutants compared with the WT and *sg1* mutant (Fig. 7A–D). The levels of soluble proteins were profiled in 2-week-old Col, *sg1*, *gun1*, *sg1 gun1*, *gun4*, and *sg1 gun4* plants (with cotyledons removed). Consistent with the observations of the alleviated phenotypes, the amounts of RbcL and RbcS in the double mutant plants were significantly higher than were those in *sg1*, but lower than were those in the WT (Fig. 7E). Subsequently the *sg1 gun5* double mutant was constructed, and it was revealed that the mutation of *GUN5* (with a function similar to that of *GUN1* and *GUN4* in retrograde signalling) did not alleviate the *sg1* phenotype. On the contrary, it slightly enhanced the albino phenotypes of *sg1* (see Supplementary Fig. S3 available at *JXB* online). Taken together, the present results indicate that the *gun1* and *gun4* mutations in *sg1* may partially restore the disordered expression patterns of chloroplast relative genes, thereby alleviating the *sg1* phenotypes.

## Discussion

TPR-containing proteins comprise a common group of proteins that participate in protein–protein interactions or assembly of multiprotein complexes. The TPR domain consists of a degenerate, 34 amino acid sequence, which is present in tandem arrays of 3–16 motifs (D’Andrea and Regan, 2003; Whitfield and Mainprize, 2010). The TPR proteins have been found to be involved in many diverse processes within eukaryotic cells, including synaptic vesicle fusion (Young *et al.*, 2003), peroxisomal targeting and import (Brocard and Hartig, 2006; Fransen *et al.*, 2008), and mitochondrial and chloroplast import (Baker *et al.*, 2007; Mirus *et al.*, 2009). In the present study, a novel chloroplast-localized TPR-containing protein, *SG1*, was identified which is required for chloroplast development.

### Influence of TPR protein SG1 mutation on normal chloroplast development

A large number of TPR-containing proteins are predicted to target to either mitochondria or chloroplasts (Small and Peeters, 2000). Consistent with their localization, many TPR proteins, for example pTAC2, Nac2, Toc64, Pyg7, and LAP1, have been reported to be involved in chloroplast development (Boudreau *et al.*, 2000; Sohrt and Soll, 2000; Peng *et al.*, 2006; Pfalz *et al.*, 2006; Stockel *et al.*, 2006; Kalanon and McFadden, 2008). These TPR proteins regulate chloroplast development in many ways, including gene expression (pTAC2), mRNA processing (Nac2), and protein transport and assembly (Toc64, Pyg7, and LAP1). SG1 is also required for chloroplast development; however, the mechanism of regulation differs from those of previously reported TPR proteins. First, and most importantly, SG1 is only required for the early stage of chloroplast development; once the plant has grown, the chloroplast becomes normal, and the seedling can grow photoautotrophically. In contrast, many previously reported TPR mutations, such as *ptac2* and *pyg7*, are lethal (Pfalz *et al.*, 2006; Stockel *et al.*, 2006). Secondly, previously reported TPR protein regulation of chloroplast gene expression showed high specificity; for example, pTAC2 directly regulates the expression of PEP genes (Pfalz *et al.*, 2006). In contrast, the *sg1* mutant showed disrupted expression of PEP genes, NEP genes, nuclear-encoded chloroplast genes, and chlorophyll biosynthesis genes. It could not be determined whether SG1 was involved in mRNA processing, protein transport, or assembly. However, the inconsistency between the mRNA levels and protein levels of RbcL and RbcS in the *sg1* mutant suggests that *SG1* may be involved in protein biosynthesis or degradation in chloroplast development.

The present protein alignment data indicated that the mutation site of SG1 is located within the first TPR motif; this site is not a conserved site in the motif, but is conserved in its homologous sequences in *Arabidopsis* (see Supplementary Fig. S2B available at *JXB* online). The mutation may not affect the protein–protein interaction scaffolds formed by the TPR motif, but affect the proper function of SG1 in chloroplast development. The gene analysis showed that the expression of chloroplast-related genes was changed in the process of the *sg1* albino leaf becoming green; for example, *RbcL* and *accD* which were decreased in albino leaf were up-regulated, while *rpoB* which was increased in albino leaf was down-regulated (Fig. 5A–D). These changes may re-establish a new balance among the chloroplast-related genes in the *sg1* mutant and result in the leaf turning green. Further studies should be conducted to clarify the mechanisms by which SG1 regulates chloroplast development.

### Mutation of *GUN1* and *GUN4* ameliorating *sg1* phenotypes

The chloroplast developmental status has been shown to control a set of nuclear genes that encode chloroplast-localized proteins via a process known as retrograde signalling (Surpin *et al.*, 2002). *GUN* genes (including *GUN1*–*GUN6*) are important for sending signals to the nucleus, to regulate nuclear-encoded chloroplast gene expression (Susek *et al.*, 1993). In the present study, the *gun1* and *gun4* mutations ameliorated the slow-greening phenotypes of *sg1* (Fig. 6A), and the leaves of *sg1 gun1* and *sg1 gun4* double mutants showed higher chlorophyll contents than did the leaves of *sg1* mutant plants, at all growth stages (see Supplementary Table S1 available at *JXB* online). These results indicate that GUN1 and GUN4 genetically interact with SG1. GUN1 and GUN4 are both important factors in the retrograde signalling pathway of chloroplast development. Therefore, to investigate whether other components of retrograde signalling interact with SG1, the *sg1 gun5* double mutant was constructed, and it was revealed that *sg1 gun5* did not alleviate the *sg1* phenotypes (see Supplementary Fig. S3 available at *JXB* online). Possible explanations are that GUNs play very important but differing roles in retrograde signalling; SG1 genetically interacts with GUN1 and GUN4 through their differing roles from GUN5 in retrograde signalling; or that the mutation of GUN1 and GUN4 ameliorates *sg1* phenotypes through other roles in chloroplast development, apart from their involvement in the retrograde signalling pathway.

The present gene expression data revealed that *gun1* and *gun4* mutation in plants with the *sg1* background altered the disturbed expression pattern of chloroplast-related genes in *sg1*, which may partially restore the imbalanced expression of chloroplast-related genes caused by *sg1* mutation. The expression changes brought by *gun1* and *gun4* are not identical, and were therefore capable of ameliorating *sg1* phenotypes to different degrees. Further, the results of protein analysis showed that the contents of RbcL and RbcS in *sg1 gun1* and *sg1 gun4* double mutants were significantly higher than were those in the *sg1* mutant. The abundance of RbcL and RbcS was higher in *sg1 gun1* than in *sg1 gun4* (Fig. 7E), possibly indicating the different degrees to which *gun1* and *gun4* restore the phenotypes of *sg1*. Taken together, the results indicate that *gun1* and *gun4* can partially restore the imbalance of chloroplast-related genes in *sg1*, thereby alleviating the defective phenotypes.

## Supplementary data

Supplementary data are available at *JXB* online.

Figure S1. Early growth of *sg1* favoured by sucrose, and delayed embryogenesis during *sg1* seed development.

Figure S2. Phylogenetic analysis and amino acid sequence alignment of SG1 TPR domains.

Figure S3. The phenotypes of the *sg1 gun5* double mutant.

Table S1. Chlorophyll contents of leaves from different genotypes at different growth stages.

Table S2. The segregated ratio of different phenotypic seedlings in the F_1_ offspring of a reciprocal cross between Col and *sg1*.

Table S3. Primers for markers used in first mapping.

Table S4. Primers for markers used for fine mapping.

Table S5. Primers for qRT–PCR.

Supplementary Data
