# Supplementary material for: The tetratricopeptide repeat-containing protein slow green1 is required for chloroplast development in Arabidopsis
Source: J Exp Bot. 2014 Jan 13;65(4):1111–23. doi: 10.1093/jxb/ert463 (PMC3935568; doi:10.1093/jxb/ert463)
Supplement: Supplementary Data [file supp_65_4_1111__index.html]

The tetratricopeptide repeat-containing protein slow green1 is required for chloroplast development in Arabidopsis — The tetratricopeptide repeat-containing protein slow green1 is required for chloroplast development in Arabidopsis — Supplementary Data 

# The tetratricopeptide repeat-containing protein slow green1 is required for chloroplast development in *Arabidopsis*

## Supplementary Data

Data files

**Files in this Data Supplement:**

- Supplementary Data - Supplementary Data
- Supplementary Data - Supplementary Data
- Supplementary Data - Supplementary Data
- Supplementary Data - Supplementary Data
- Supplementary Data - Supplementary Data
