# Supplementary material for: The tetratricopeptide repeat-containing protein slow green1 is required for chloroplast development in Arabidopsis
Source: J Exp Bot. 2014 Jan 13;65(4):1111–23. doi: 10.1093/jxb/ert463 (PMC3935568; doi:10.1093/jxb/ert463)
Supplement: Supplementary Data [file supp_ert463_jexbot107888_file001.pdf]

Journal of Experimental Botany

The Tetratricopeptide Repeat-Containing Protein Slow Green1 Is Required for Chloroplast Development in *Arabidopsis*

Zhihong Hu, Fan Xu, Liping Guan, Pingping Qian, Yaqiong Liu, Huifang Zhang, Yan Huang, and Suiwen Hou

Supplementary Information

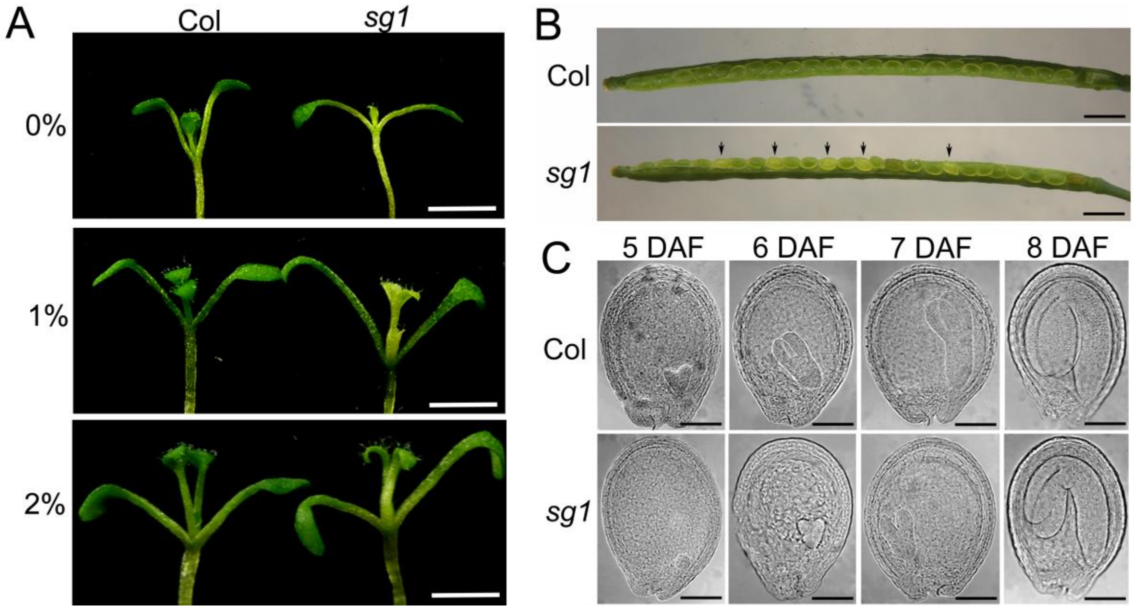

Figure S1. Early growth of *sg1* favored by sucrose, and delayed embryogenesis during *sg1* seed development. (A) 10 days old seedlings grew on Murashige–Skoog medium supplemented with 1% and 2% sucrose. (B) 10 DAF siliques from wild and *sg1*$^{+/-}$ heterozygous. (C) Embryo development in wild-type (upper panels) and *sg1* plants (lower panels). Arrowheads showed the seeds of delayed embryogenesis. Abbreviations: DAF, day after fertilization. Bars = 0.5 cm in (A); 0.5 mm in (B); and 100 μm in (C).

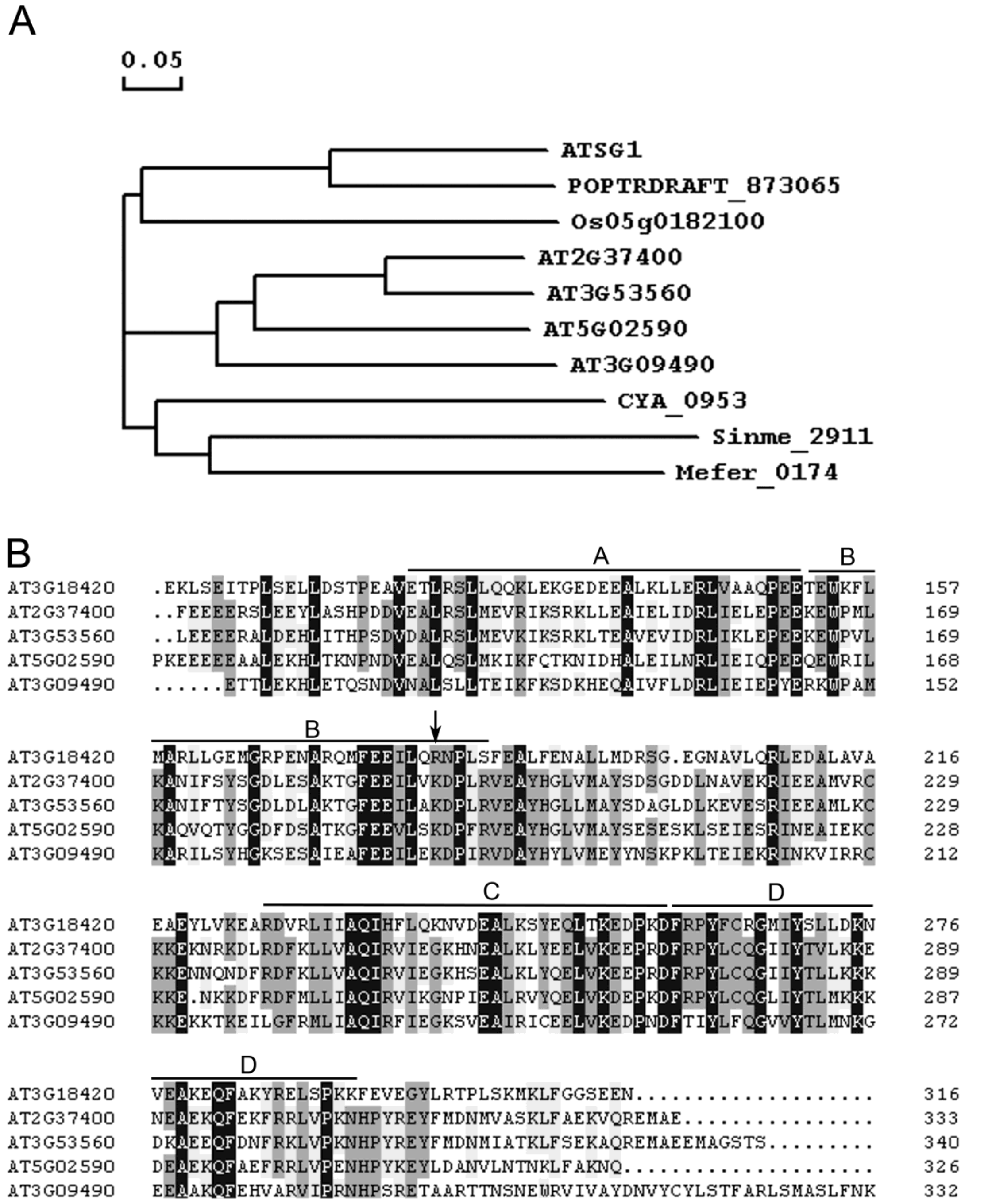

Figure S2. Phylogenetic analysis and amino acid sequence alignment of SG1 TPR domains. (A) Unrooted phylogenetic tree of SG1 and homologous proteins in *Arabidopsis thaliana*, *Populus trichocarpa*, *Oryza sativa*, *Synechococcus*, *Sinorhizobium* and *Methanocaldococcus*. (B) Comparison of the amino acid sequence of SG1 with *Arabidopsis thaliana* proteins AT3G09490, AT2G37400, AT3G53560, and AT5G02590. Black boxes indicate strictly conserved amino acids among these proteins. The arrow head indicates the mutation site of *sg1*; bars above the sequences labelled A to D indicate the four TPR domains of SG1.

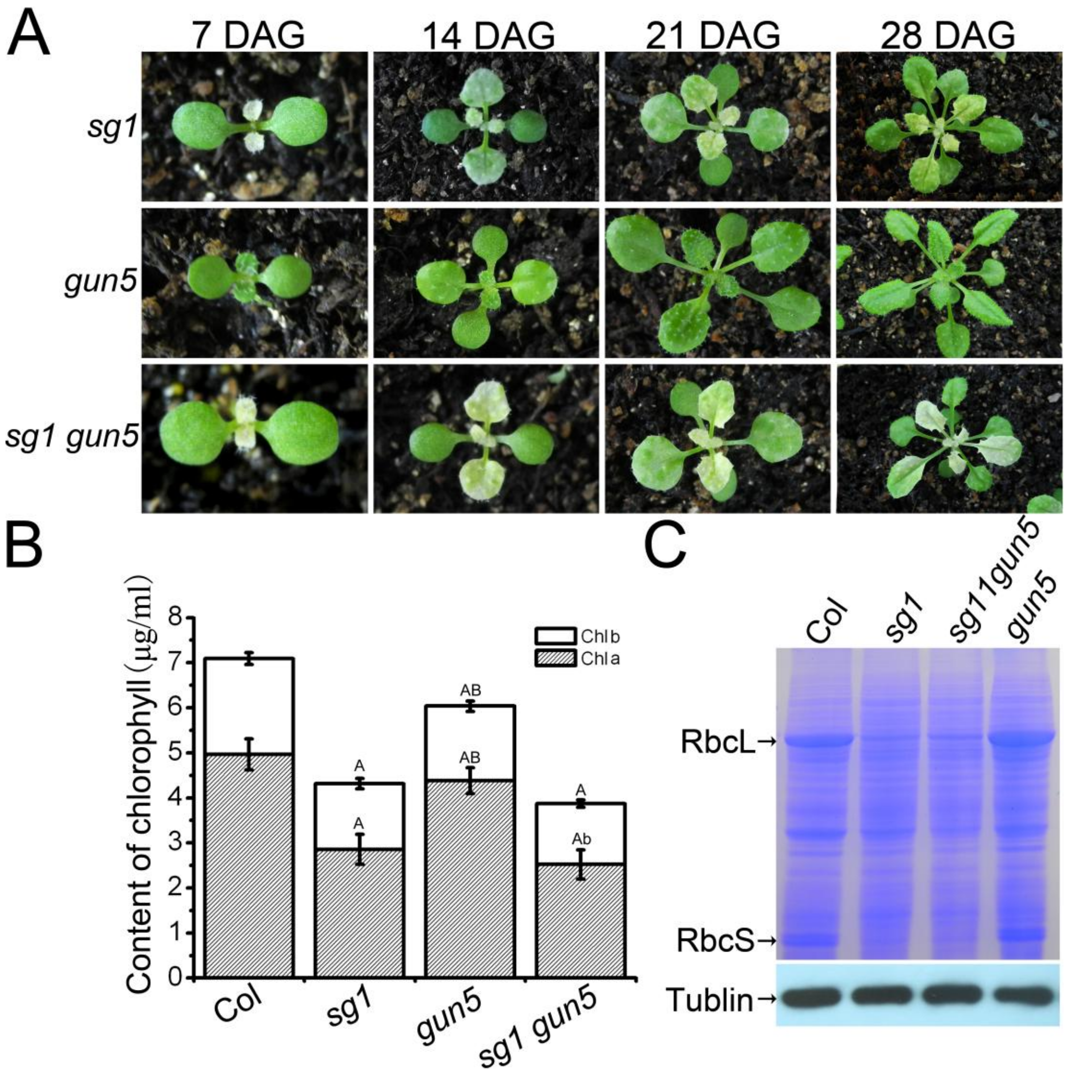

Figure S3. The phenotypes of *sg1 gun5* double mutant. (A) The seedlings of *sg1*, *gun5* and *sg1 gun5* grown for 1 to 4 weeks in soil. (B) Chlorophyll contents in 2-week-old Col, *sg1*, *gun5*, and *sg1 gun5* plants. (C) Total protein resolved by SDS-PAGE from Col, *sg1*, *sg1 gun5* and *gun5*, the arrows indicate the major bands of the large subunit (RbcL) and small subunit (RbcS) of RuBisCO. [a] Significantly different from Col, $P < 0.05$; [A] Significantly different from Col, $P < 0.01$; [b] Significantly different from *sg1*, $P < 0.05$; [B] Significantly different from *sg1*, $P < 0.01$.

Tables

Supplementary Table S1. Chlorophyll contents of leaves from different genotypes at different growth stages. Total chlorophylls were obtained from the sixth basal rosette leaves of 3-week-old, 4-week-old, and 5-week-old plants. Values represent mean ± SD of three independent experiments. [a] Significantly different from Col, P<0.05, [A] Significantly different from Col, P<0.01, [b] Significantly different from *sg1*, P<0.05, [B] Significantly different from *sg1*, P<0.01.

Table 1. Chlorophyll contents of Col, *sg1, gun1*, *sg1 gun1*, *gun4,* and *sg1 gun4* leaves (μg/mL)

		3 weeks	4 weeks	5 weeks		3 weeks	4 weeks	5 weeks
	Col	3.08±0.135	3.19±0.076	3.27±0.085		1.33±0.046	1.34±0.084	1.39±0.075
	sg1	0.87±0.037[A]	1.70±0.084[A]	2.66±0.066[A]		0.434±0.049[A]	0.82±0.059[A]	1.18±0.085[A]
Chl *a*	*gun1*	2.97±0.015[aB]	3.06±0.047[B]	3.17±0.043[B]	Chl *b*	1.20±0.032[aB]	1.23±0.043[B]	1.32±0.054[B]
	sg1 gun1	2.24±0.053[AB]	2.90±0.042[aB]	3.10±0.015[aB]		1.09±0.016[AB]	1.25±0.024[aB]	1.41±0.038[aB]
	gun4	1.52±0.126[AB]	1.76±0.11[AB]	2.22±0.078[AB]		0.55±0.07[AB]	0.67±0.067[AB]	0.81±0.081[AB]
	sg1 gun4	1.77±0.083[AB]	2.15±0.091[AB]	2.59±0.112[AB]		0.96±0.072[AB]	1.07±0.044[AB]	1.14±0.065[AB]

Supplementary Table S2. The number of different phenotypic seedlings segregated in the F1 offspring of a reciprocal cross between Col and *sg1*. Seedlings of 10-day-old were examined to gather statistics. X^2 was calculated based on an expected ratio of 3:l.

Cross	Number of seedlings		Approximate ratio of wild/mutant	X^2
	Wild-type	Mutant		
sg1 (♂)× Col (♀)	249	79	3:1	0.14 (P>0.5)
Col (♂) × *sg1* (♀)	171	58	3:1	0.01 (P>0.5)

Supplementary Table S3. Primers of markers for first mapping.

Chromosome	Markers	Forward（5'→3'）	Reverse（5'→3'）
1	EAT	GCCACTGCGTGAATGATATG	CGAACAGCCAACATTAATTCCC
	JV18I19	AATTCAGTATCGAGATACCCCTCT	TGTCGTATATCAATCGAAAAAGAGAT
	NGA392	TTGAATAATTTGTAGCCATG	GGTGTTAAATGCGGTGTTC
	NGA280	CTGATCTCACGGACAATAGTGC	GGCTCCATAAAAAGTGCACC
2	NGA1145	CCTTCACATCCAAAACCCAC	GCACATACCCACAACCAGAA
	NGA1126	CGCTACGCTTTTCGGTAAAG	GCACAGTCCAAGTCACAACC
	NGA168	TCGTCTACTGCACTGCCG	GAGGACATGTATAGGAGCCTCG
3	AthBIO2b	TGACCTCCTCTTCCATGGAG	TTAACAGAAACCCAAAGCTTTC
	NGA172	AGCTGCTTCCTTATAGCGTCC	CATCCGAATGCCATTGTTC
	NGA162	CATGCAATTTGCATCTGAGG	CTCTGTCACTCTTTTCCTCTGG
	GAPAb	CACCATGGCTTCGGTTACTT	TCCTGAGAATTCAGTGAAACCC
4	NGA6	TGGATTTCTTCCTCTCTTCAC	ATGGAGAAGCTTACACTGATC
	NGA8	GAGGGCAAATCTTTATTTCGG	TGGCTTTCGTTTATAAACATCC
	NGA1139	TAGCCGGATGAGTTGGTACC	TTTTTCCTTGTGTTGCATTCC
	NGA1107	GCGAAAAAACAAAAAAATCCA	CGACGAATCGACAGAATTAGG
5	NGA225	GAAATCCAAATCCCAGAGAGG	TCTCCCCACTAGTTTTGTGTCC
	NGA106	GTTATGGAGTTTCTAGGGCACG	TGCCCCATTTTGTTCTTCTC
	NGA76	GGAGAAAATGTCACTCTCCACC	AGGCATGGGAGACATTTACG
	NGA129	TCAGGAGGAACTAAAGTGAGGG	CACACTGAAGATGGTCTTGAGG

Supplementary Table S4. Primers of markers for fine mapping

Chromosome	Markers	Forward（5’→3’）	Reverse（5’→3’）
3	T11I18	GAAGGATATTTCCCCAATTCG	GACCCCTACCCGCTCCTTGAC
	MSJ11	TAAGTGCATATATCCAGTAC	CATTCTTCAGTTCTTGTATG
	MDC8	TCCACTCATGAATCGTATTG	ACTATGACTATCCATCCATG
	MKP6	ATCTTGTAATCTGGTGCGTG	GCAATAGCCCATGTGAATTC
	MYF24.3	GAAATGTCGTCATCTTGTTC	TGTATAACTTCCTCCACAAG
	MYF24	ACGATAATGTATGTGTGTAC	TCAGTCTTCCTTACAATATG
	MIE15	TCTCTGTAAATTTCTCCAGC	GTTGAGAAGCATACTAGAGA

Supplementary Table S5. Primers of qRT-PCR

Name	Forward（5’→3’）	Reverse（5’→3’）
ß-tubulin	GATTTCAAAGATTAGGGAAGAGTA	GTTCTGAAGCAAATGTCATAGAG
SG1	GCTGAGGCTGAGTATTTGGTGA	GCTCTTCAATGCTTCATCCAC
psaB	GGACCCCACTACTCGTCGTA	ATTGCTAATTGCCCGAAATG
psbA	GAGCAGCAATGAATGCGATA	CCTATGGGGTCGCTTCTGTA
psbB	CGTGCGACTTTGAAATCTGA	TAGCACCATGCCAAATGTGT
RbcL	GTGTTGGGTTCAAAGCTGGT	CATCGGTCCACACAGTTGTC
accD	TGTGGATTCAATGCGACAAT	TTTTGCGCAGAGTCAATACG
ycf2.2	GGGTTCAACAAGCAATCGAT	CCGATGTATCATTTCTGGGT
rpoA	CAAGCCGACACAATAGGCAT	AGCGCGTTGCGCGTTCCAT
PsbO	CTCGAAACTTCCTCGGCTC	AATCCGGCGATTTTGACAG
PORB	GCAATGCTGCGGTTTATTTC	GAGAAGAAAATGTCCCAAATGG
rpoB	GCGAAAGAATCCTCCTATGC	CCACCTCACATCAATAACTC
CAB2	TCGCAAGGAACCGTGAGCTA	AGCCTTGAACCAAACTGCCT
RbcS	CCTAGACCCTCCGATCACTC	GGTTTGGTCTAGTGCTTTGG

METHODS USED IN SUPPLEMENTARY MATERIALS

Observation of embryo development

To examine the development of the embryos, ovules from the self-fertilising heterozygous siliques at different developmental stages were treated with a clearing solution of chloral hydrate, water, and glycerol (8:3:1, w/v/v) for 12 h. The developing embryos were then analysed under an optical microscope (Motic).
