# Supplementary figures and images for: The tetratricopeptide repeat-containing protein slow green1 is required for chloroplast development in Arabidopsis
Source: J Exp Bot. 2014 Jan 13;65(4):1111–23. doi: 10.1093/jxb/ert463 (PMC3935568; doi:10.1093/jxb/ert463)

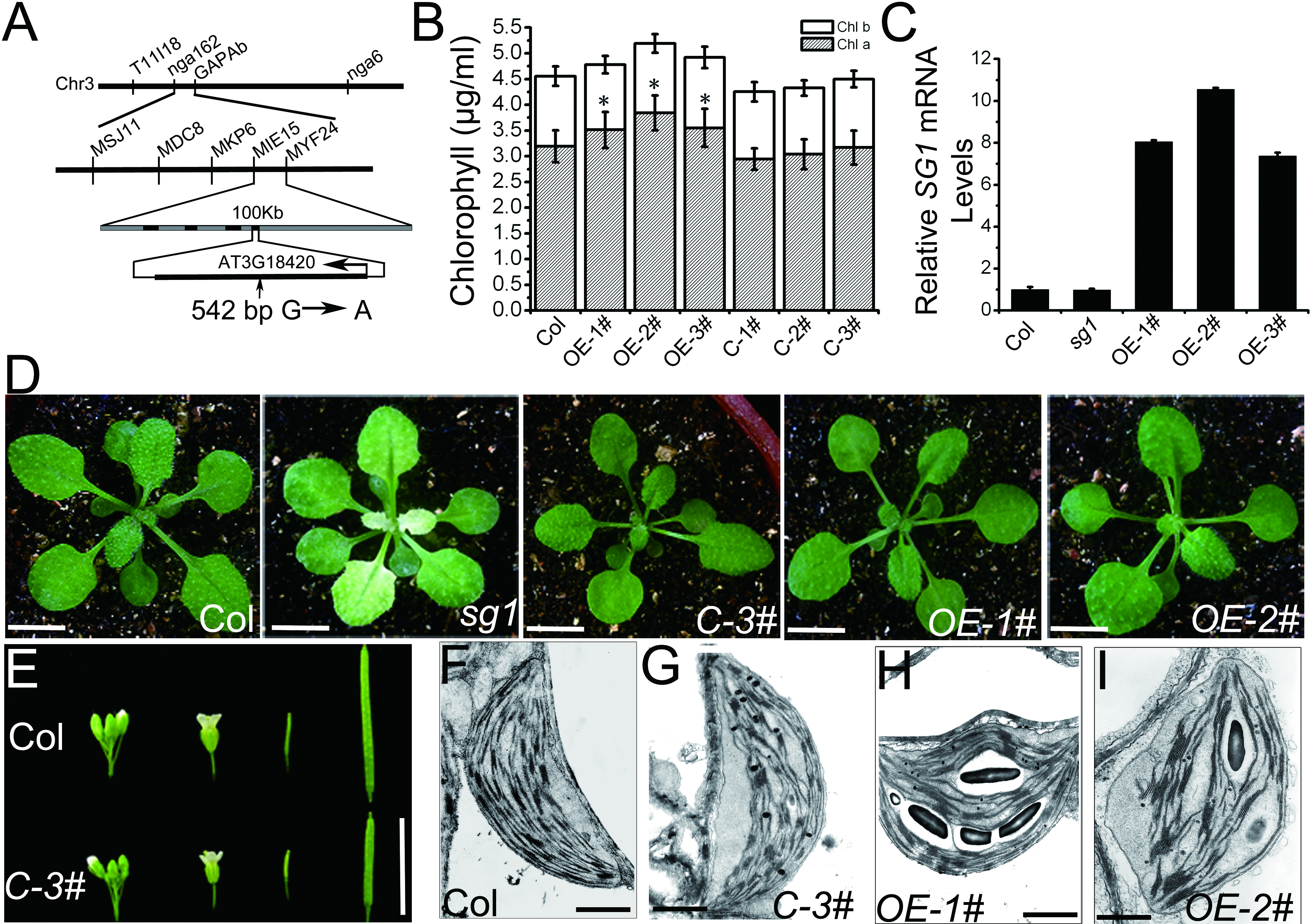

Supplement: Supplementary Data [file supp_ert463_jexbot107888_file003.tif]

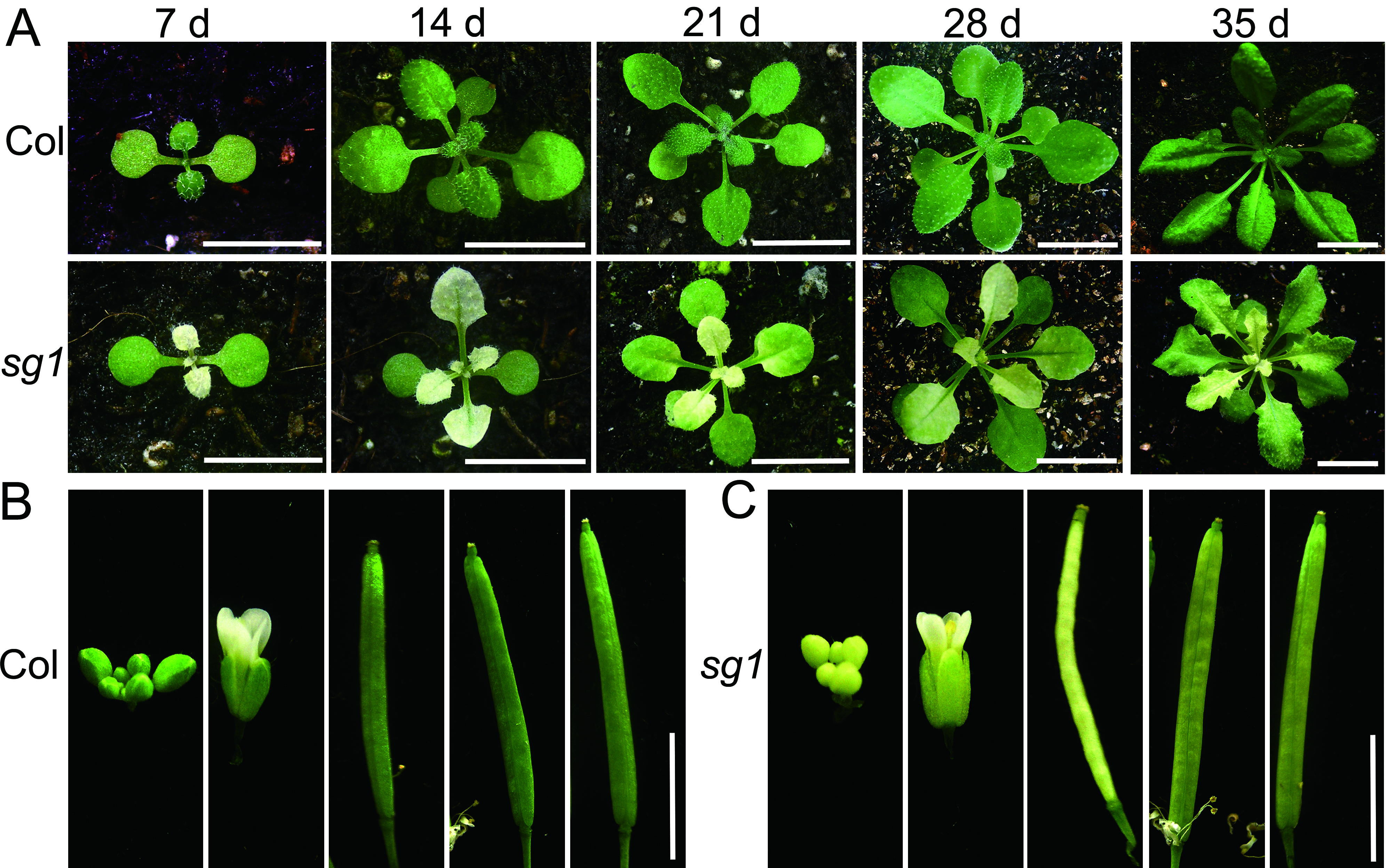

Supplement: Supplementary Data [file supp_ert463_jexbot107888_file002.tif]

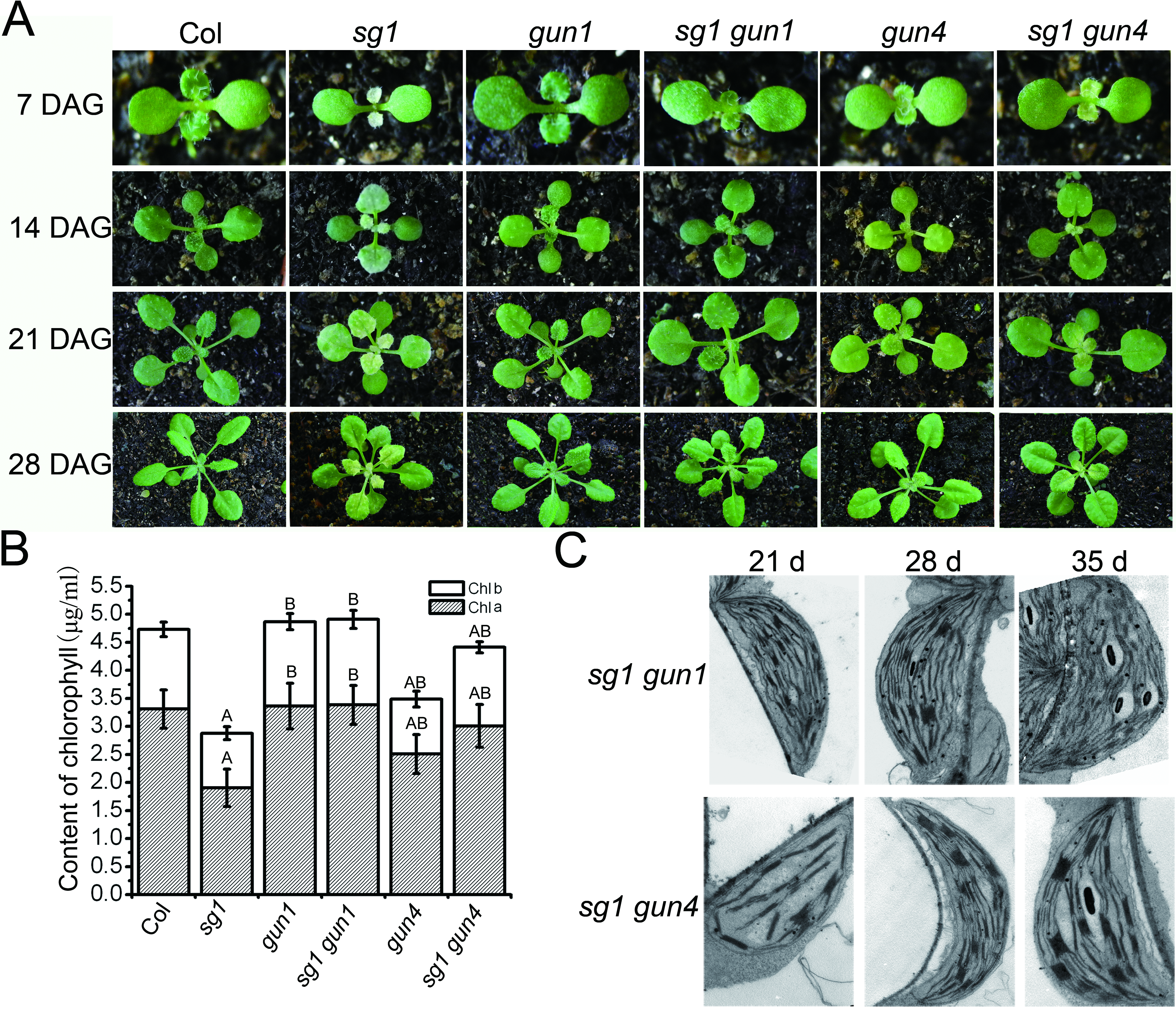

Supplement: Supplementary Data [file supp_ert463_jexbot107888_file005.tif]
